# UMG1 Defines a Targetable Subset of T‐Cell Lymphomas and Enables Precision Immunotherapy With a First‐in‐Class CD3ε Bispecific Engager

**DOI:** 10.1002/hon.70187

**Published:** 2026-03-15

**Authors:** Daniele Caracciolo, Carlo Gentile, Sara Squillacioti, Stefania Signorelli, Caterina Riillo, Pinuccia Faviana, Francesco Conforti, Katia De Ieso, Elisabetta Procopio, Emanuela Altomare, Nicoletta Polerà, Maria Gaetano, Estelle Balducci, Omer Beganovic, Franca Maria Tuccillo, Patrizia Bonelli, Katia Grillone, Ludovic Lhermitte, Pierosandro Tagliaferri, Pierfrancesco Tassone

**Affiliations:** ^1^ Department of Experimental and Clinical Medicine (DMSC) Magna Graecia University Catanzaro Italy; ^2^ Department of Surgical Medical Molecular Pathology and Critical Area University of Pisa Pisa Italy; ^3^ Pathology Unit Annunziata Hospital Cosenza Italy; ^4^ Pathology Unit A.O.U Dulbecco Catanzaro Italy; ^5^ IRIB‐CNR Catanzaro Italy; ^6^ Laboratory of Onco‐Hematology Assistance Publique‐Hôpitaux de Paris Hôpital Necker Enfants‐Malades Paris France; ^7^ Molecular Biology and Viral Oncology Unit Istituto Nazionale Tumori—IRCCS—Fondazione Pascale Napoli Italy

**Keywords:** bispecific T‐cell engagers, cancer, immunotherapy, lymphoma, T‐cell lymphomas, T‐PLL, UMG1

## Abstract

T‐cell lymphomas (TCLs) account for a relatively small fraction of lymphoid malignancies and are characterized by highly aggressive course often refractory to current available therapies. We previously reported potent in vitro and in vivo antitumor activity of a Bispecific T‐Cell Engager (UMG1/CD3ε‐BTCE) directed against UMG1, a unique CD43 epitope that is abundantly expressed on T‐cell acute lymphoblastic leukemia (T‐ALL) and diffuse large B‐cell lymphoma (DLBCL) cells, while absent in most normal tissues, except thymocytes and a small fraction of peripheral blood T lymphocytes (< 5%). Here, we investigated the in vitro efficacy of UMG1/CD3ε‐BTCE against TCLs. IHC analysis of Tissue Micro Arrays (TMAs) revealed high UMG1 expression in 62.3% of TCL samples, including peripheral T‐cell lymphoma‐not otherwise specified (PTCL‐NOS) and ALK‐negative anaplastic large cell lymphoma (ALCL). Notably, all T‐PLL primary specimens (27/27) were positive, and 3 of 4 TCL cell lines also expressed UMG1 by flow cytometry. The asymmetric UMG1/CD3ε‐BTCE induced robust redirected cytotoxicity against UMG1‐expressing TCL cells. Moreover, this activity was strengthened by cell exposure to the HDAC inhibitor SAHA. We observed a dose‐dependent engaged T‐cell‐mediated cytotoxicity and inflammatory cytokine release, resulting in lysis of UMG1‐expressing cells, with no significant effect on UMG1‐not expressing cells. Our findings suggest that the UMG1/CD3ε‐BTCE selectively exerts potent anti‐tumor activity against a relevant subset of TCLs. These findings support the development of a precision immunotherapy approach for patients with UMG1‐expressing aggressive hematologic malignancies.

T‐cell lymphomas (TCLs) are rare and aggressive hematologic malignancies that include approximately 10%–15% of non‐Hodgkin lymphomas. They include highly heterogeneous histologic entities, molecular profiles, and poor clinical outcomes, particularly in the relapsed or refractory setting [1].

In this context, UMG1, a cancer‐associated epitope of CD43, represents a high promising therapeutic target. Unlike the canonical pattern of CD43 expression, which is broadly found across a wide variety of normal hematopoietic cells, the UMG1 epitope exhibits a highly restricted distribution, limited to cortical thymocytes and a small subset of peripheral T lymphocytes (< 5%). No other normal cells/tissues express UMG1. This restricted tissue specificity opens the possibility for selective immune targeting while minimizing on‐target/off‐tumor toxicity [2, 3]. Indeed, UMG1‐directed monoclonal antibodies and bispecific T‐cell engagers (BTCEs) previously demonstrated selective cytotoxicity in T‐acute lymphoblastic leukemia (T‐ALL) [4] and diffuse large B‐cell lymphoma (DLBCL) [5], supporting UMG1 translational potential as agnostic target.

Building on this rationale, we tested the expression of UMG1 across TCLs and assessed the cytotoxic and immune‐modulatory activities of asymmetric 2 + 1 IgG‐like UMG1/CD3ε‐BTCE in both cell lines and primary patient samples.

Immunohistochemistry (IHC) on tissue microarrays (TMAs) revealed that UMG1 was expressed in 62.3% (38/61) of TCLs, with more than 50% of malignant cells showing strong membrane staining in the majority of positive cases (Figure 1A, *left*), including 46.1% (18/39) of peripheral T‐cell lymphoma not otherwise specified (PTCL‐NOS) and 85% (6/7) of ALK‐negative anaplastic large cell lymphomas (ALCL) (Figure 1A, *right*). Among primary samples, UMG1 was detected in 8 out of 10 diagnostic tissue sections, including Mycosis Fungoides and rare Primary Cutaneous Peripheral T‐cell Lymphoma (Figure 1B) (Table S1). Most notably, all tested T‐prolymphocytic leukemia (T‐PLL) samples (27/27) expressed UMG1, with a variable ratio (UMG1/IgG1 negative control) of median fluorescence intensities (MFI) (ranging from 8.5 to 3149), indicating a consistent targetability of this aggressive and rare leukemia subtype (Figure 1C–D) (Table S2).

**FIGURE 1:**
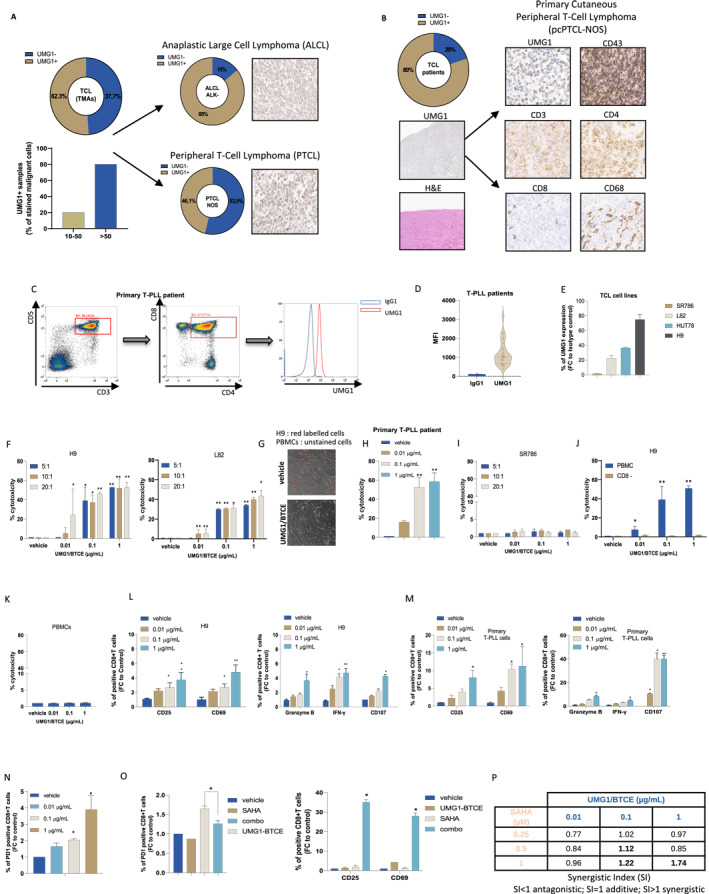
UMG1/CD3ε‐BTCE redirects and activates T lymphocytes against TCL cells. (A) *Left.* Percentage of UMG1 membrane expression observed on TCL samples included in TMA (*top*). Percentage of UMG1‐expressing lymphoma cells among stained TMA cases (*bottom*). *Right.* Percentage of UMG1‐expression and representative IHC images, among ALK negative ALCL (*top*) and PTCL NOS (*bottom*) TMA cases. (B) Percentage of UMG1 positive samples from TCL pathology slides and representative IHC analysis of patient with Primary cutaneous peripheral T cell lymphoma. UMG1 and H&E staining (20x) and UMG1, CD43, CD3, CD4, CD8, and CD68 staining (DAB chromogen reaction, 40X) are shown. (C) Flow cytometry analysis performed on T‐PLL cells from a UMG1 positive patient. (D) MFI analysis of UMG1 T‐PLL samples (*n* = 27) as compared to isotype control (IgG1). (E) Flow cytometry analysis of UMG1 expression on TCL cell lines. Fold change (FC) to Isotype control (IC) is reported. (F) Redirected T‐cell‐mediated lysis monitored by viable target of H9 and L82 (UMG1+) CellTrace Violet‐labeled cells, co‐cultured with PBMCs at different E:T ratios (20:1, 10:1 e 5:1) and treated for 48 h with increasing UMG1/CD3ε‐BTCE concentrations (0.01, 0.1 and 1 μg/mL). Then target and effector cells were stained with 7‐AAD and redirected T cell‐mediated cytotoxicity was evaluated by flow cytometry as 7‐AAD+/Violet‐positive cells (%). (G) Coupling of T‐cells on H9 cells by UMG1/CD3ε‐BTCE (1 μg/mL), as assessed by immunofluorescence microscopy: H9 cells were stained with CellTrace Far Red. (H) Redirected T‐cell‐mediated lysis monitored by viable target of primary UMG1+ T‐PLL cells co‐cultured with PBMCs at a 10:1 of E:T ratio and treated for 48 h with increasing UMG1/CD3ε‐BTCE concentrations. (I) SR786 (UMG1‐) CellTrace Violet‐labeled cells, co‐cultured with PBMCs at different E:T ratios (20:1, 10:1,5:1) and treated for 48 h with increasing UMG1/CD3ε‐BTCE concentrations. Target and effector cells were stained with 7‐AAD and redirected T cell‐mediated cytotoxicity was evaluated by flow cytometry as 7‐AAD+/Violet‐positive cells (%). (J) H9 target cells were co‐cultured with healthy donor derived PBMCs with or without immunomagnetic CD8+ T cells depletion in the presence of 0.01, 0.1 and 1 μg/mL of UMG1/CD3ε‐BTCE or vehicle. Cytotoxicity (%) of H9 cells is shown. (K) PBMCs were treated in the presence of 0.01, 0.1 and 1 μg/mL of UMG1/CD3ε‐BTCE or vehicle. Cytotoxicity (%) of PBMC is shown. (L) H9 cells were co‐cultured for 48 h with PBMCs (E:T = 10:1) in the presence of increasing doses of UMG1/CD3ε‐BTCE. Percentages of CD69‐and CD25‐positive (left), granzyme B‐, IFN‐γ and CD107‐ (right) positive CD8+ T cells are shown. (M) UMG1+ Primary T‐PLL cells were co‐cultured for 48 h with PBMCs (E:T = 10:1) in the presence of increasing doses of UMG1/CD3ε‐BTCE. Percentages of CD69‐and CD25‐positive (left), granzyme B‐, IFN‐γ and CD107‐ (right) positive CD8+ T cells are shown. (N) H9 cells were co‐cultured for 48 h with PBMCs (E:*T* = 10:1) in the presence of UMG1/CD3ε‐BTCE or vehicle. Flow cytometric analysis of PD1 expression on CD8+ T cells is shown. (O) H9 cells were co‐cultured for 48 h with PBMCs (E:T = 10:1) in the presence of UMG1/CD3ε‐BTCE or vehicle, with or without SAHA. Flow cytometric analysis of PD1 (left) and activation markers CD25 and CD69 (right) on CD8+ T cells are shown. (P) Table showing synergistic indexes resulting from combinatorial treatments of H9 with UMG1/CD3ε‐BTCE and SAHA (48 h time point). Student's *t*‐test was applied to calculate statistical significance: *p < 0.05; **p < 0.01. BTCE, bispecific T‐cell engager; FC, fold change; IHC, immunohistochemistry; MFI, Median of Fluorescence Intensity; PBMC, peripheral blood mononuclear cell. TCL, T‐cell lymphoma; TMA, tissue microarray.

UMG1 expression was also confirmed in 3 out of 4 TCL cell lines: H9 (high), HUT78 and L82 (low), while SR‐786 were UMG1‐negative (Figure 1E).

Importantly, UMG1/CD3ε‐BTCE treatment induced dose‐dependent cytotoxicity selectively against UMG1+ targets. H9 and L82 cell lines exhibited significant cell death, with up to 60% lysis of primary T‐PLL cells (Figure 1F–H), while no significant cytotoxicity was detected in UMG1‐ cells or in the absence of effector CD8 T cells (Figure 1I–J), confirming antigen‐specific and T cell‐dependent killing. Most importantly, our results show that UMG1/CD3ε‐BTCE did not induce significant cytotoxicity in PBMCs, indicating no fratricide effects (Figure 1K).

UMG1/CD3ε‐BTCE treatment led to robust T‐cell activation, demonstrated by CD69 and CD25 increased expression on CD8+ T lymphocytes, CD107a degranulation marker upregulation, and granzyme B and IFN‐γ secretion. These effects were observed both in cell lines and in primary patient‐derived specimens (Figure 1L–M). UMG1/CD3ε‐BTCE thus demonstrates effective immune synapse formation and cytolytic function, translating antigen engagement into effective tumor cell killing.

Of note, a subset of T cells engaged by UMG1/CD3ε‐BTCE exhibited increased expression of exhaustion markers, including PD‐1 (Figure 1N), suggesting that prolonged stimulation might induce a negative regulatory phenotype. To overcome this counteracting effect, we investigated the activity of UMG1/CD3ε‐BTCE in combination with histone deacetylase inhibitor (HDACi) SAHA (vorinostat), which has demonstrated immune‐sensitizing properties and is approved for relapsed Cutaneous and Peripheral T‐cell Lymphoma (CTCL and PTCL) [6].

Pre‐treatment of PBMCs with sub‐lethal doses of SAHA decreased UMG1/CD3ε‐BTCE‐induced exhaustion marker upregulation, enhanced activation of CD8+ T‐cell, and increased cytotoxicity against H9 cells (Figure 1O–P). Notably, since H9 cells exhibit constitutive UMG1 expression exceeding 90%, and sub‐lethal doses of SAHA were employed to avoid direct intrinsic cytotoxicity, the increased anti‐tumor activity appeared driven by enhanced T‐cell effector function, providing a rationale for combining UMG1/CD3ε‐BTCE with epigenetic modulators to overcome T‐cell exhaustion mechanisms and prolong therapeutic responses in TCLs.

The therapeutic index of bispecific antibodies targeting T‐cell malignancies remains a critical concern. Previous strategies targeting pan‐T‐cell markers, like CD4, CD5, CD7, and TCR constant regions, suffer significant challenges, including T‐cell aplasia and fratricide effects [7, 8]. In contrast, the restricted expression profile of UMG1 provides a safe therapeutic window. Additionally, the asymmetric 2 + 1 UMG1/CD3ε‐BTCE architecture ensures lower activation of circulating T cells, extending half‐life and improving tolerability.

The clinical implications of our research are substantial. T‐PLL remains one of the most difficult‐to‐treat leukemias, with a median overall survival of less than 1 year after relapse and few therapeutical options [9]. The extensive expression of UMG1 in T‐PLL strongly supports its use as a therapeutic biomarker and candidate for targeted and personalized intervention. Moreover, despite the high histologic heterogeneity of TCL, approximately 60% of tested TMAs and 80% of diagnostic tissues, including ALK negative ALCL and PTCL‐NOS, showed positive staining for UMG1. Importantly, our data indicate that UMG1 act as onco‐fetal antigen appearing during thymic ontogeny [4], being silenced in mature T‐cells, and re‐emerging upon malignant transformation, suggesting UMG1 as a stable marker linked to malignant transformation.

These results establish UMG1/CD3ε‐BTCE as a first‐in‐class candidate for immunotherapy of TCLs. Its favorable target profile, strong cytotoxic activity, and amenability to biomarker‐driven patient selection suggest potential applications in different treatment lines. Considering the challenges of CAR T‐cell manufacturing [10], especially in T‐cell neoplasms, BTCEs offer a practical and off‐the‐shelf alternative with rapid translational potential.

In summary, our study identifies UMG1 as a relevant tumor‐associated target significantly expressed in a variety of TCLs and demonstrates that UMG1/CD3ε‐BTCE can elicit a potent, antigen‐specific cytotoxicity through redirection of T‐cell effector functions. These findings support further development of UMG1‐directed immune therapeutics for precision approaches for these challenging and still lethal malignancies.

## Author Contributions

D.C., C.R., S.S., S.S., M.G., N.P., and E.A. performed experiments and/or analyzed the data. C.G., F.C., L.L., E.B., and O.B. provided biological samples and analyzed the data. P.T. developed the mAb. C.G., P.F., K.D.I., E.P., and F.C. performed I.H.C. analysis. D.C., C.R., F.M.T., P.B., K.G., and L.L. provided critical evaluation of experimental data and of the manuscript. D.C. and PTagliaferri and PTassone conceived the study and wrote the manuscript. PTagliaferri and PTassone supervised the study.

## Funding

This manuscript has been supported by BiovelocITA, Italy, and partially by Italian Ministry of Health PSC Salute 2014‐2020–POS4 “Cal‐Hub‐Ria” (Grant T4‐AN‐09), Fondazione Roche (Grant Fondazione Roche per la Ricerca 2024) and Italian Ministry of Health, Ricerca Corrente 2024.

## Consent

The authors have nothing to report.

## Conflicts of Interest

The authors declare no conflicts of interest.

## Supporting information


Supporting Information S1

**Table S1:** IHC analysis of primary TCL subtypes evaluated for UMG1 expression.

**Table S2:** MFI of 27 T‐PLL primary samples positive to UMG1 staining. MFI of isotype control (IgG1), and the ratio between UMG1 and IgG1 MFI are also reported.

## Data Availability

The data that support the findings of this study are available from the corresponding author upon reasonable request.
