# Supplementary material for: UMG1 Defines a Targetable Subset of T‐Cell Lymphomas and Enables Precision Immunotherapy With a First‐in‐Class CD3ε Bispecific Engager
Source: Hematol Oncol. 2026 Mar 15;44(2):e70187. doi: 10.1002/hon.70187 (PMC12989738; doi:10.1002/hon.70187)
Supplement: Supplementary file 1 — Supporting Information S1 [file HON-44-e70187-s003.docx]

**Supplementary Material and Methods**

**Immunohistochemistry (IHC)**

Lymphoma tissue microarrays (TMAs) (Catalog No: MC1081, NHL401a, LM482c, LY2084, LM242) containing 61 cases of T-cell Lymphomas (TCLs), produced by Tissuearray.com LLC, were incubated overnight with the primary antibodies UMG1, (dilution 1:300) at 4°C. The immunostaining by either a polymer detection method (Novolink Polymer Detection Systems Novocastra Leica Biosystems Newcastle Ltd Product No: RE7280-K) and AEC (3-amino-9-ethylcarbazole, Dako, Ref K3464) substrate-chromogen ready to use, was revealed.

For immunostaining of tissue sections, the samples were dewaxed and rehydrated. Novocastra Epitope Retrieval Solutions (pH9 EDTA-based buffer) was used for antigen unmasking technique which was performed in thermostatic bath [FALC Instruments S.r.L, Treviglio (BG) Italy, Model WB-MD 5] at 98°C for 30 minutes.

Next, the sections were brought at room temperature and washed in PBS, endogenous peroxidase was neutralized with 3% H_2_O_2_ for 10 minutes and a specific protein blocking solution was added for 8 minutes.

Then, slides are counterstained with Harris Hematoxylin (Novocastra, Leica Biosystems) and Ki67 (Dako, clone MIB-1; 1:150).

All the sections were observed by Zeiss Axio Scope A1 optical microscope (Zeiss, Germany) and microphotographs were collected using an Axiocam 503 Color digital camera with the ZEN2 imaging software (Zeiss Germany). Aperio CS2 Leica was used to scan the sections.

**Cell lines**

H9, HUT-78, L82 and SR-786 cells were purchased by DSMZ. Cell lines were cultured in RPMI 1640 (Gibco®, Thermo Fisher Scientific, Waltham, MA, USA), supplemented with 10% fetal bovine serum (Lonza Group Ltd., Basel, Switzerland), 100 U/mL penicillin, and 100 µg/mL streptomycin (Gibco®, Thermo Fisher Scientific) and maintained at 37 °C in a 5% CO_2_ atmosphere, as previously reported (1, 2).

**Patient samples and UMG1 expression analyses**

Peripheral blood mononuclear cells (PBMCs) from healthy donors’ were collected at the Medical Onco-Hematology Units of the Renato Dulbecco Teaching Hospital/Magna Graecia University of Catanzaro, Italy. Mononuclear cells were collected by Ficoll-Paque Plus (GE Healthcare) centrifugation and washed twice in culture medium (RPMI-1640 supplemented with 10% Fetal Bovine Serum, FBS).

T-PLL samples were collected and analysed at the Hôpital Necker Enfants-Malades of Paris Informed consent was obtained in accordance with the Declaration of Helsinki. To evaluate UMG1 expression on peripheral blood of T-PLL by standard multiparametric flow cytometry (FC), 1 μg/mL of anti-human UMG1-PE or IgG1-PE for 20 minutes at room temperature in the dark and a cocktail of conjugated monoclonal antibodies: anti-CD3, anti-CD5, Ant-CD4 and anti-CD8 (BD Bioscences). 500,000 total events for each tube were acquired on a FACScanto™ II flow cytometer (Becton Dickinson) and analyzed with Diva™ software (Becton Dickinson).

TCL cell lines were stained with 1μg/mL of anti-human UMG1-PE or IgG1-PE for 20 minutes at room temperature in the dark.

**UMG1/CD3ε-BTCE**

Humanized UMG1 mAb (huUMG1) has been generated as previously described (3). To obtain a monovalent BTCE binding UMG1 and CD3ε (UMG1/CD3ε-BTCE), “knobs-into-holes” technology was used on the basis of humanized UMG1 sequence (3).

**Redirected T-cell cytotoxicity assay**

TCL target cell lines were labeled with CellTrace Violet (Gibco®, Thermo Fisher Scientific, USA) viable marker, according to manufacturer instructions, and co-cultured with healthy donor derived PBMCs at 20:1, 10:1 e 5:1 E:T ratio, in the presence of 0.01, 0.1 and 1 µg/mL of UMG1/CD3ε-BTCE or vehicle (PBS 1x, Gibco®, Thermo Fisher Scientific, Waltham, MA, USA) for 48h at 37 °C and 5% CO_2_. Then target and effector cells were stained with 7-AAD (#51-68981E) (BD Biosciences). Redirected T cell-mediated cytotoxicity was evaluated by flow cytometry (Attune NxT Flow cytometer, Thermo Fisher Scientific) as 7-AAD+/ Violet-positive cells (%). In the cytotoxicity experiment with T-cell depletion, immunomagnetic cell sorting using CD8 microbeads (MACS Miltenyi Biotec) was performed before co-culture experiments.

**T-cell activation and proliferation**

UMG1 expressing target H9 and primary T-PLL cells were co-cultured with PBMCs at 10:1 E:T ratio for 48-72 hours at 37°C and 5% CO_2_. T cells were stained with anti-human CD8 (SK1) APC-Cy7 (#641400), CD3 (UCHT1) PerCP-Cy5.5 (#560835), CD45 (HI30) BV510 (#563204), CD25 APC (#555434), CD69 PE (#555531) (BD Biosciences), for 20 min at room temperature in the dark or with CD107a PE (#555801) (BD Biosciences, 4h of incubation). T cells were gated for CD8- positive, and for CD69, CD25, or CD107a-positive cells.

For intracellular staining, UMG1 expressing target H9 or primary T-PLL cells and PBMCs were plated at 10:1 E:T ratio in the presence of increasing concentrations of UMG1/CD3ε-BTCE, or vehicle for 72h. Brefeldin A 10 µg/mL was added, and cells were incubated at 37°C, 5% CO_2_. After 4h,cells were stained with surface antibodies as previously described, washed twice with 1X PBS, fixed with reagent A (Nordic-MUbio, Susteren, Netherlands) for 15 minutes protected from light, then washed with 1X PBS, permeabilized with Reagent B (Nordic- MUbio) and stained with anti-human IFN-γ PE (#559327), granzyme B AlexaFluor647 (#560212), for 15 minutes at room temperature protected from light. After incubation, samples were washed in 1X PBS and analyzed by ATTUNE NxT flow cytometer (Thermo Fisher Scientific).

**UMG1/CD3ε-BTCE combinatorial approach and T-cell exhaustion**

PBMCs from healthy donors were pre-treated with increasing concentrations of SAHA (Sigma Aldrich). 24h after, H9 CellTrace Violet-labeled cells were seeded in a 24-well plate and co-cultured with PBMCs at 10:1 E:T ratio with increasing concentrations of UMG1/CD3ε-BTCE at 37°C, 5% CO_2_. After 48h of treatment, cells were collected, stained with 7-AAD and analyzed by flow cytometry. Exhaustion markers were evaluated by multiparametric flow cytometry panel on CD8+T-cells by anti-human CD45 BV510, CD3 PerCP-Cy5.5, CD8 (SK1) APC-Cy7 (#641400) and PD-1 PE-Cy7 (#561272) purchased from BD Biosciences.

**Immunofluorescence analysis**

H9 cells were labeled with CellTrace Far Red (Thermo Fisher Scientific), according to manufacturer instructions, co-cultured at 10:1 E:T ratio and incubated with 1 μg/mL UMG1/CD3ε-BTCE. Samples were then washed twice with 1X PBS, spotted with cytospin technique to microscope slides, fixed by 4% PFA in 1X PBS, and finally wet-mounted for confocal microscopic imaging using Leica SP8.

**Statistical analysis**

Each experiment was performed at least three times and values are reported as means ± SD. Graphs were obtained using Graphpad Prism version 9.4.1. p value of less than 0.05 was accepted as statistically significant. The synergistic index was determined as previously described (4, 5).

1. Caracciolo D, Di Martino MT, Amodio N, Morelli E, Montesano M, Botta C, et al. miR-22 suppresses DNA ligase III addiction in multiple myeloma. Leukemia. 2019;33(2):487-98.

2. Caracciolo D, Juli G, Riillo C, Coricello A, Vasile F, Pollastri S, et al. Exploiting DNA Ligase III addiction of multiple myeloma by flavonoid Rhamnetin. J Transl Med. 2022;20(1):482.

3. Caracciolo D, Riillo C, Ballerini A, Gaipa G, Lhermitte L, Rossi M, et al. Therapeutic afucosylated monoclonal antibody and bispecific T-cell engagers for T-cell acute lymphoblastic leukemia. J Immunother Cancer. 2021;9(2).

4. Gulla A, Di Martino MT, Gallo Cantafio ME, Morelli E, Amodio N, Botta C, et al. A 13 mer LNA-i-miR-221 Inhibitor Restores Drug Sensitivity in Melphalan-Refractory Multiple Myeloma Cells. Clinical cancer research : an official journal of the American Association for Cancer Research. 2016;22(5):1222-33.

5. Caracciolo D, Riillo C, Juli G, Scionti F, Todoerti K, Polera N, et al. miR-22 Modulates Lenalidomide Activity by Counteracting MYC Addiction in Multiple Myeloma. Cancers (Basel). 2021;13(17).
