# Supplementary material for: UMG1 Defines a Targetable Subset of T‐Cell Lymphomas and Enables Precision Immunotherapy With a First‐in‐Class CD3ε Bispecific Engager
Source: Hematol Oncol. 2026 Mar 15;44(2):e70187. doi: 10.1002/hon.70187 (PMC12989738; doi:10.1002/hon.70187)
Supplement: Supplementary file 2 — Table S1: IHC analysis of primary TCL subtypes evaluated for UMG1 expression. [file HON-44-e70187-s002.pptx]

## Slide 1
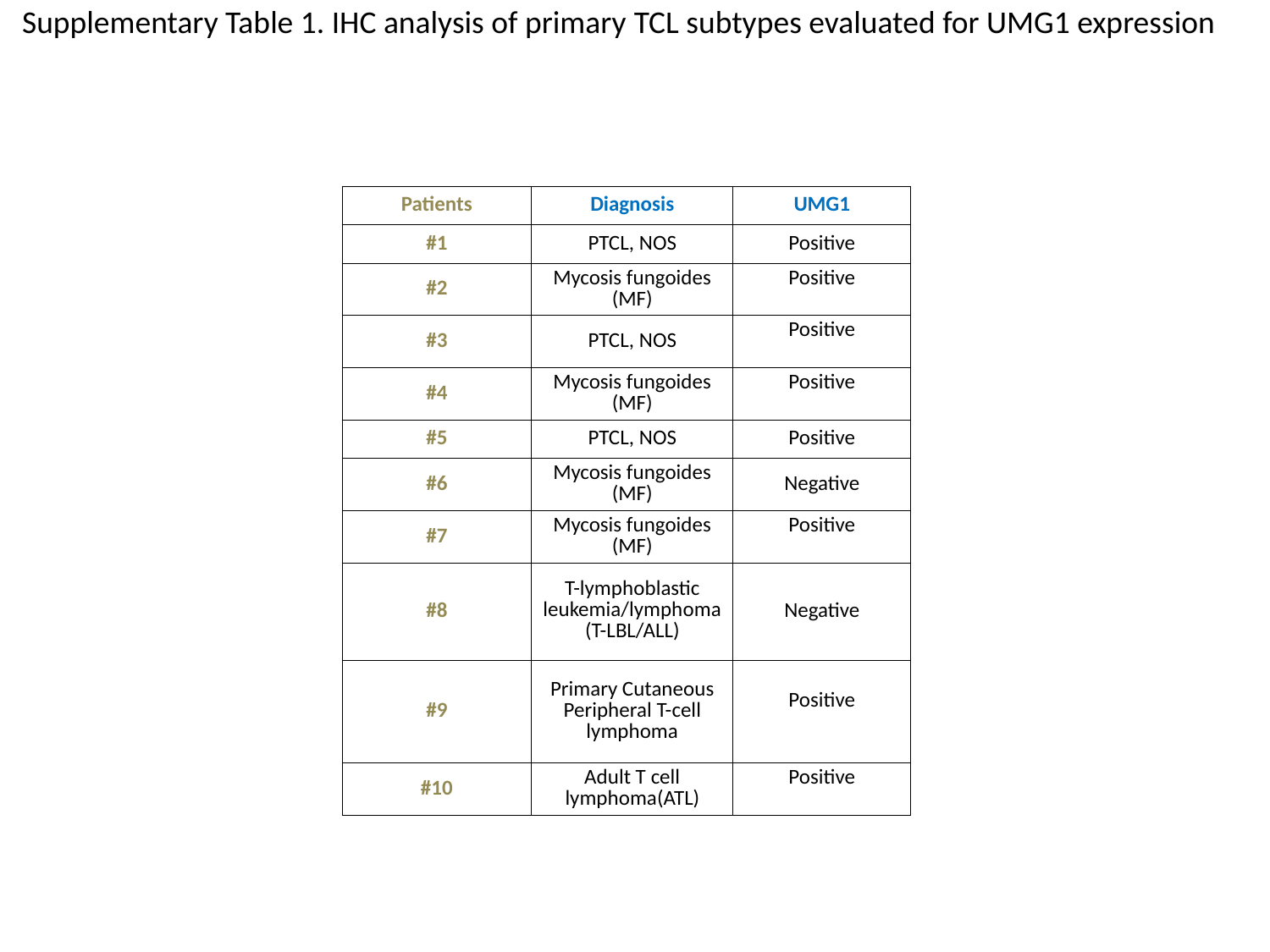

Supplementary Table 1. IHC analysis of primary TCL subtypes evaluated for UMG1 expression
| Patients | Diagnosis | UMG1 |
| --- | --- | --- |
| #1 | PTCL, NOS | Positive |
| #2 | Mycosis fungoides (MF) | Positive |
| #3 | PTCL, NOS | Positive |
| #4 | Mycosis fungoides (MF) | Positive |
| #5 | PTCL, NOS | Positive |
| #6 | Mycosis fungoides (MF) | Negative |
| #7 | Mycosis fungoides (MF) | Positive |
| #8 | T-lymphoblastic leukemia/lymphoma (T-LBL/ALL) | Negative |
| #9 | Primary Cutaneous Peripheral T-cell lymphoma | Positive |
| #10 | Adult T cell lymphoma(ATL) | Positive |
