# Supplementary material for: UMG1 Defines a Targetable Subset of T‐Cell Lymphomas and Enables Precision Immunotherapy With a First‐in‐Class CD3ε Bispecific Engager
Source: Hematol Oncol. 2026 Mar 15;44(2):e70187. doi: 10.1002/hon.70187 (PMC12989738; doi:10.1002/hon.70187)
Supplement: Supplementary file 3 — Table S2: MFI of 27 T‐PLL primary samples positive to UMG1 staining. MFI of isotype control (IgG1), and the ratio between UMG1 and IgG1 MFI are also reported. [file HON-44-e70187-s001.pptx]

## Slide 1
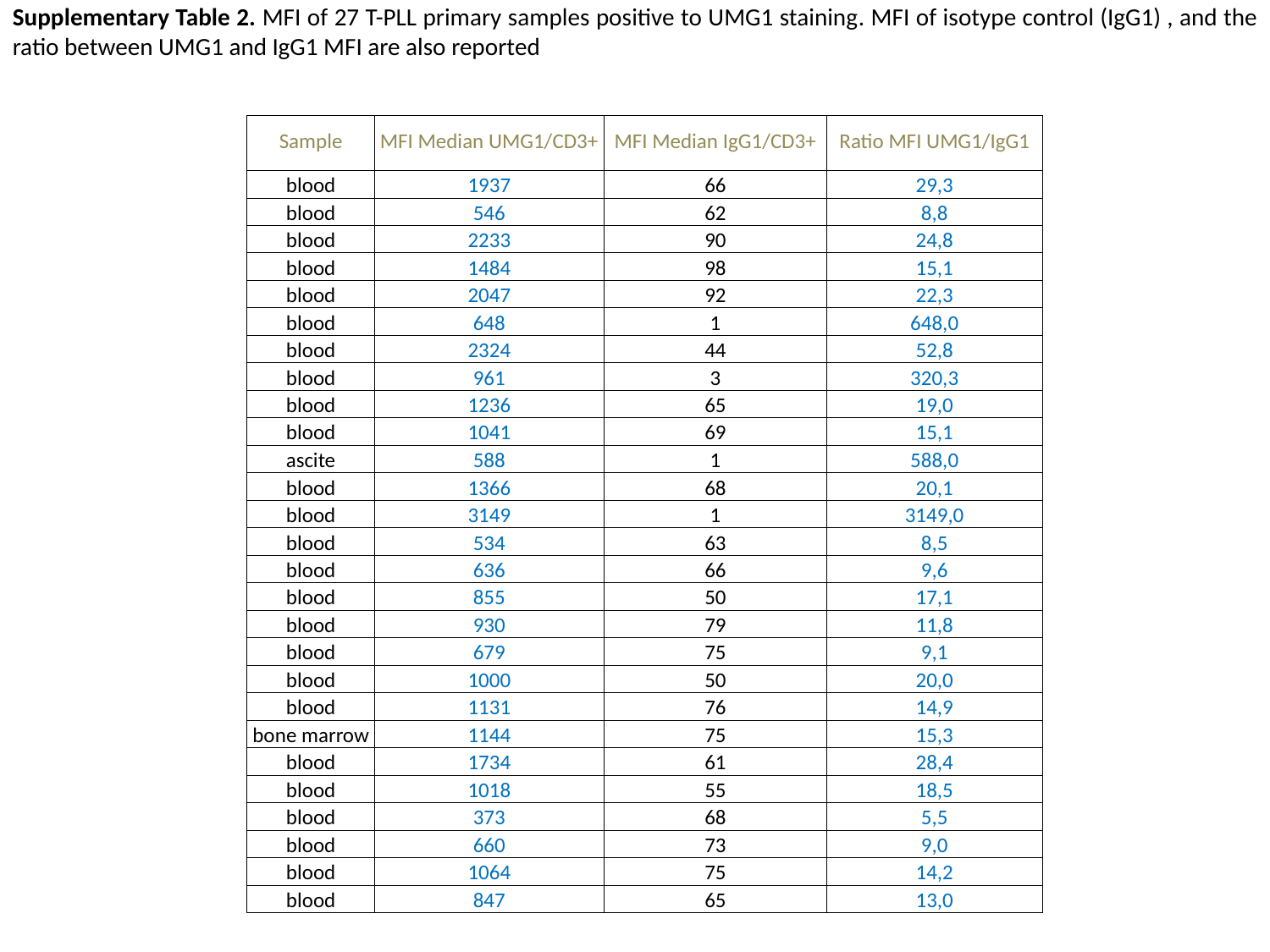

Supplementary Table 2. MFI of 27 T-PLL primary samples positive to UMG1 staining. MFI of isotype control (IgG1) , and the ratio between UMG1 and IgG1 MFI are also reported
| Sample | MFI Median UMG1/CD3+ | MFI Median IgG1/CD3+ | Ratio MFI UMG1/IgG1 |
| --- | --- | --- | --- |
| blood | 1937 | 66 | 29,3 |
| blood | 546 | 62 | 8,8 |
| blood | 2233 | 90 | 24,8 |
| blood | 1484 | 98 | 15,1 |
| blood | 2047 | 92 | 22,3 |
| blood | 648 | 1 | 648,0 |
| blood | 2324 | 44 | 52,8 |
| blood | 961 | 3 | 320,3 |
| blood | 1236 | 65 | 19,0 |
| blood | 1041 | 69 | 15,1 |
| ascite | 588 | 1 | 588,0 |
| blood | 1366 | 68 | 20,1 |
| blood | 3149 | 1 | 3149,0 |
| blood | 534 | 63 | 8,5 |
| blood | 636 | 66 | 9,6 |
| blood | 855 | 50 | 17,1 |
| blood | 930 | 79 | 11,8 |
| blood | 679 | 75 | 9,1 |
| blood | 1000 | 50 | 20,0 |
| blood | 1131 | 76 | 14,9 |
| bone marrow | 1144 | 75 | 15,3 |
| blood | 1734 | 61 | 28,4 |
| blood | 1018 | 55 | 18,5 |
| blood | 373 | 68 | 5,5 |
| blood | 660 | 73 | 9,0 |
| blood | 1064 | 75 | 14,2 |
| blood | 847 | 65 | 13,0 |
